# Evaluation of the Manchester COVID-19 Urgent Eyecare Service (CUES)

**DOI:** 10.1038/s41433-021-01522-0

**Published:** 2021-04-30

**Authors:** Rahul Kanabar, Wendy Craven, Helen Wilson, Rebecca Rietdyke, Felipe Dhawahir-Scala, Matthew Jinkinson, William D. Newman, Robert A. Harper

**Affiliations:** 1 Manchester Medical School, The University of Manchester, Oxford Road, Manchester, M13 9PL UK; 2 Primary Eyecare Service, 2.3 Waulk Mill, 51 Bengal Street, Manchester, M4 6LN UK; 3 Manchester Royal Eye Hospital and Manchester Academic Health Sciences Centre, Manchester University NHS Foundation Trust Manchester, Manchester, M13 9WL UK; 4 Division of Pharmacy and Optometry, School of Health Sciences, Faculty of Biology, Medicine and Health, University of Manchester, Manchester, M13 9PL UK

**Keywords:** Education, Eye diseases, Health services, Outcomes research

## Abstract

**Introduction:**

Pressure on capacity in ophthalmology alongside the coronavirus (COVID-19) pandemic led to the development of the COVID-19 Urgent Eyecare Service (CUES), allowing patients to receive a prompt ophthalmic consultation, including remotely. The aim of this study was to conduct a service evaluation of CUES in Manchester.

**Methods:**

Data were collected both prospectively and retrospectively from both primary and secondary care over an 8-week period from June to August 2020.

**Results:**

In primary care CUES in Greater Manchester (GM) 2461 patients were assessed, with a majority self-referring to the service (68.7%, *n* = 1844). 91.7% of cases initially screened for CUES were deemed eligible and given a telemedicine appointment in GM; 53.3% of these cases required face-to-face consultation. 14.3% of cases seen within in GM CUES (351 out of 2461) were provisionally referred to secondary care. Contemporaneously the main provider emergency eyecare department (EED) attendances were reduced by 37.7% per month between April and December 2020 inclusive, compared to the same months in 2019. Patients attending a CUES face-to-face assessment were more likely to have a diagnosis in agreement with secondary care, compared to patients referred in from telemedicine assessment only (*P* < 0.05).

**Conclusion:**

This evaluation of CUES demonstrates a high level of primary care activity alongside a sustained reduction in EED cases. The case-mix of patients seen within EED following referral appears to be of a less benign nature than those cases seen prior to the introduction of CUES.

## Introduction

In response to the disruption caused to ophthalmology by the coronavirus (COVID-19) pandemic, the COVID-19 Urgent Eyecare Service (CUES) was recommended nationally in April 2020. This new service was developed by NHS England and NHS Improvement (NHSE-I), the Local Optical Committee Support Unit (LOCSU), and the Clinical Council for Eye Health Commissioning (CCEHC). CUES aimed to allow patients to receive a prompt consultation remotely, reducing the need for conventional face-to-face consultations which would potentially endanger patients and health care professionals during the pandemic. Overall, the intended benefit of CUES was to reduce ophthalmology attendances in the HES and eye-related general practice (GP) appointments, allowing HES clinicians to focus on more urgent eye-care cases, and initially at least to contribute to the frontline efforts within critical care relating to COVID-19.

The pressing need for a service such as CUES to be introduced within eyecare pathways was evident long before the pandemic. Ophthalmology was under increasing pressure to transform services, with more cases needing to be managed in primary care, as presented in ‘The way forward’ document published by the Royal College of Ophthalmologists [1]. The longer-term need for creating capacity in ophthalmology was, therefore, already evident and COVID-19 pushed this transformation to occur with more urgency, resulting in the patient pathway jointly developed by NHSE-I, LOCSU and CCEHC [2].

The development and implementation of CUES in Manchester have been described by Harper et al. [3]. In summary, the Manchester and Greater Manchester (GM) areas are served by primary eyecare services (PES), who integrated the use of a new platform, called OPERA (optometric electronic referral and assessment), into primary care optometric practices. This IT system was linked directly with the e-referral system (eRS) and PACS in hospitals, allowing referrals to be easily made, with notifications within both primary and secondary care. CUES in Manchester was described as also capitalising on the growing number of optometrists trained and accredited with independent prescribing (IP), in theory allowing Manchester CUES to accommodate a broader case-mix of patients requiring urgent eyecare [3].

For many years prior to COVID-19 there have been extended primary care optometry services in place within the NHS in England. Developments in eye-care pathways within primary care to reduce demand in secondary care have arguably been most enduring and developed within glaucoma services for example [4]; however, in terms of more acute eye-care presentations, the Minor Eye Conditions Service (MECS), or the previously termed Primary Eye-care Assessment and Referral Service (PEARS), represent primary care services offering patients an alternative to presenting to HES A&E units for the assessment and management of some acutely presenting eye conditions. Like CUES, the aim of MECS is to manage as many patients as possible in primary care. However, the service specification for MECS does not include remote consultations and appointments are face-to-face with an optometrist [5]. The optometrist would then devise a management plan, ranging from advice and self-management to possible referral to HES. An evaluation of a South London based MECS by Konstantakopoulou et al. established that MECS produced a reduction in HES attendances, showing the potential foundation for CUES in demonstrating that referral systems can be streamlined and work efficiently [6]. While CUES can somewhat be likened to this service, CUES is focussed on more urgent eyecare and within the context of the pandemic and requirements to promote telemedicine whenever feasible. A previous study by Mas-Tur et al. explored optometric referrals to an emergency ophthalmology department; their results showed that a large proportion of patients were managed solely with advice, highlighting that referral efficiency could be improved [7]. The use of optometrists with IP accreditation and remote consultations in CUES could cut the number of HES referrals managed with advice only, potentially reducing the increasing strain on NHS emergency eyecare services [8]. CUES is now becoming very widely established [9]. However, to date, there has been no published evaluation of CUES. The NHSE-I service specification called for CUES to be evaluated and to our knowledge, this report is the first evaluation of its kind. The aim was to evaluate CUES, generically in Greater Manchester for primary care activity and then in both Manchester and Trafford specifically in respect of secondary care activity where MREH is the main provider hospital and where we were able to collate referral data.

## Methods

**Fig. 1:**
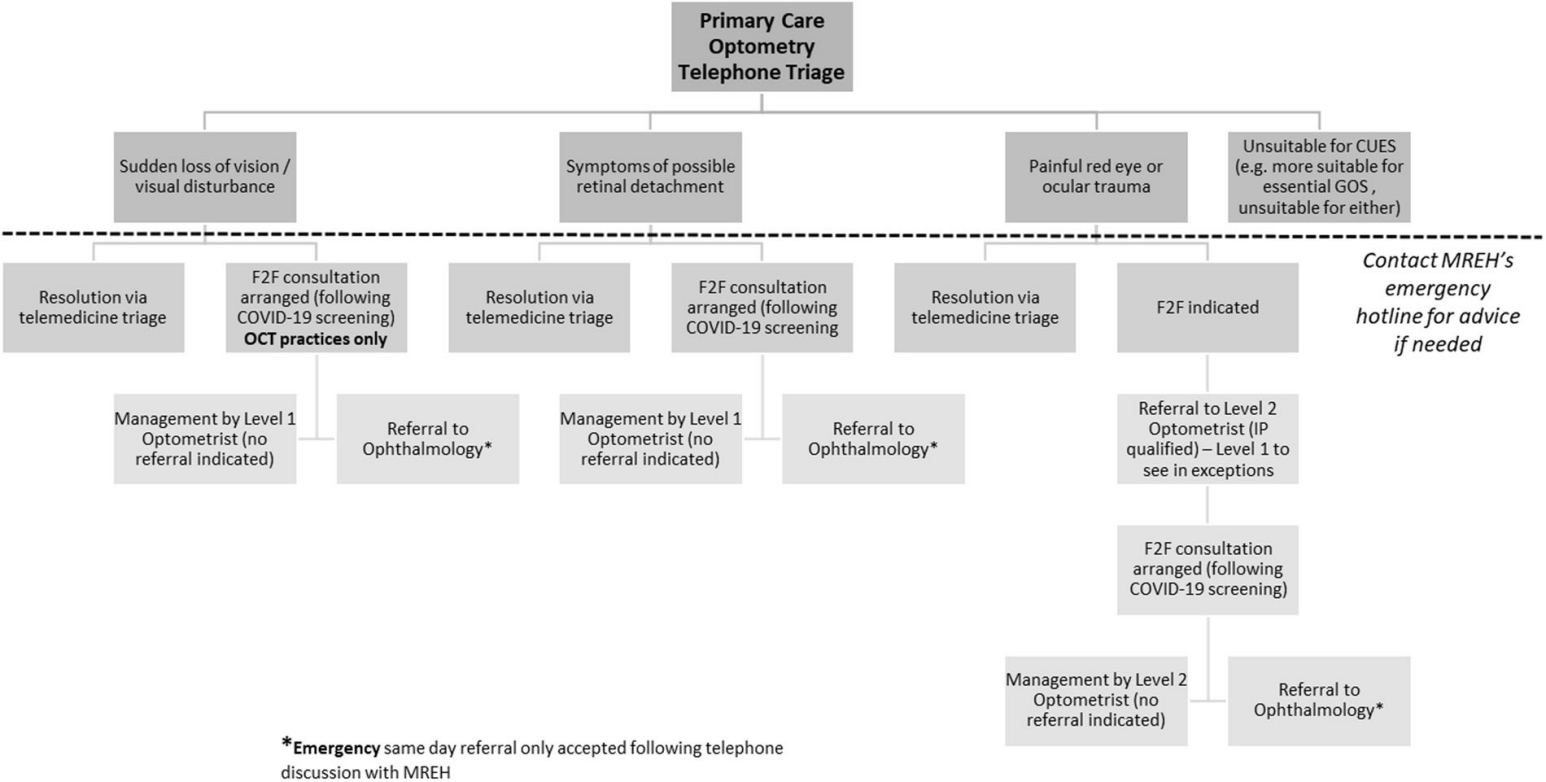
Summary clinical pathway for the Manchester CUES. F2F: Face to face consultation; GOS: General Ophthalmic Services; Level 2 optometrist has the Independent Prescribing, IP, qualification.

The CUES pathway is summarised in Fig. 1, with development and implementation summarised elsewhere [3]. The overall period of data collection for the present evaluation spanned from June 2020 to August 2020; primary care and secondary care data collection periods spanned separate dates described below. Owing to the potential for classification of patients seen within different parts of the CUES pathway over time, patients within the CUES evaluation will be categorised descriptively as follows: ‘Case’—patients assessed in primary care CUES; ‘provisional referral’ —a referral from primary care CUES to the HES that has not yet been accepted or rejected; ‘accepted referral’ - a referral from primary care CUES to the HES that has been accepted into the EED; and ‘rejected referral’ - a referral from primary care CUES to the HES that was not accepted into the EED. This manuscript adheres to SQUIRE (Standards for Quality Improvement Reporting Excellence) 2.0 guidelines.

### Primary care data

Primary care data collection was undertaken in GM over the course of ~8 weeks from 1st June to 31st July 2020 and will also be separately reported for that subset of GM CUES cases seen within the Trafford and Manchester CCGs (i.e. CUES areas referring their cases to MREH as the provider hospital and where relatively contemporaneous secondary care data was able to be evaluated). Primary care data collected included: general patient characteristics; screening outcomes and symptoms; and outcome and diagnosis at telemedicine, face-to-face (with or without the OCT arm of clinical assessment available within CUES), and follow-up appointments.

### Secondary care data

For the secondary care aspect of the evaluation, the period of 8 weeks from 17th June to 11th August 2020 was chosen, with data being collected from all CUES provisional referrals made to MREH during this time. In order to measure the quality of CUES referrals received by MREH, each provisional referral was inspected. Details from all provisional referral forms received by the MREH were recorded from each part of the referral form; data points included patient details, assessment details, symptoms at screening, provisional diagnosis, and outcome of assessment). For each CUES accepted referral, the optometrist’s diagnosis, was recorded from the referral form and the following data was recorded from the patient’s EED triage notes: date seen in EED; triage comments; hospital diagnosis/diagnoses and hospital management. Once obtained, the optometrist’s diagnosis and hospital clinician’s diagnosis for each accepted referral were compared by two experienced hospital clinicians at a consensus meeting. Each referral was assigned an accuracy rating as ‘agreement’, ‘partial agreement’ or ‘disagreement’. Partial agreement for diagnoses may have related to an optometrist recognising the diagnosis, but not entirely correctly (e.g. vitelliform macular dystrophy in the case of wet age-related macular degeneration). To further explore rejected CUES referrals (i.e. with advice and guidance) to MREH, patients’ records were searched retrospectively for each of these referrals using the patient NHS number and it was noted whether the patient subsequently attended the EED within 10 days of the date of the rejected referral. Furthermore, we retrospectively collected data for MREH EED attendances for each month from January 2019 to December 2020 inclusive. Finally, we also collated data on phone calls received by the triaging nursing team within EED from patients during 22nd July to 11th August 2020 inclusive, logging the date of calls and actions taken. In every case where the phone call had been signposted to a CUES optometrist, extra data was obtained; this included the time of phone call and symptoms of the patient. Data were only obtained for phone calls with the following actions – advised to see CUES optometrist; advised to attend MREH EED; or advised to attend local hospital/optometrist practice (e.g. Rochdale, Stepping hill, etc.). Phone calls received from optometrists to the CUES phone line in the MREH EED were recorded separately.

Microsoft Excel was used to collate all data from both primary and secondary care and assist with all analyses, largely descriptively and in line with approaches to service evaluation. Where appropriate, nonparametric statistical analysis was conducted using a chi-squared test of independence.

### Ethical considerations

Since this CUES evaluation was deemed to be a service evaluation observing the clinical pathway without any intervention there was no requirement for ethical review. The University of Manchester’s online ethics review tool as well as that of the HRA confirmed this status of our evaluation [10, 11] and within this framework, the project was registered as such within Manchester University NHS foundation Trust’s audit department (Project reference number 9130).

## Results

### Primary care optometry CUES activity

**Fig. 2:**
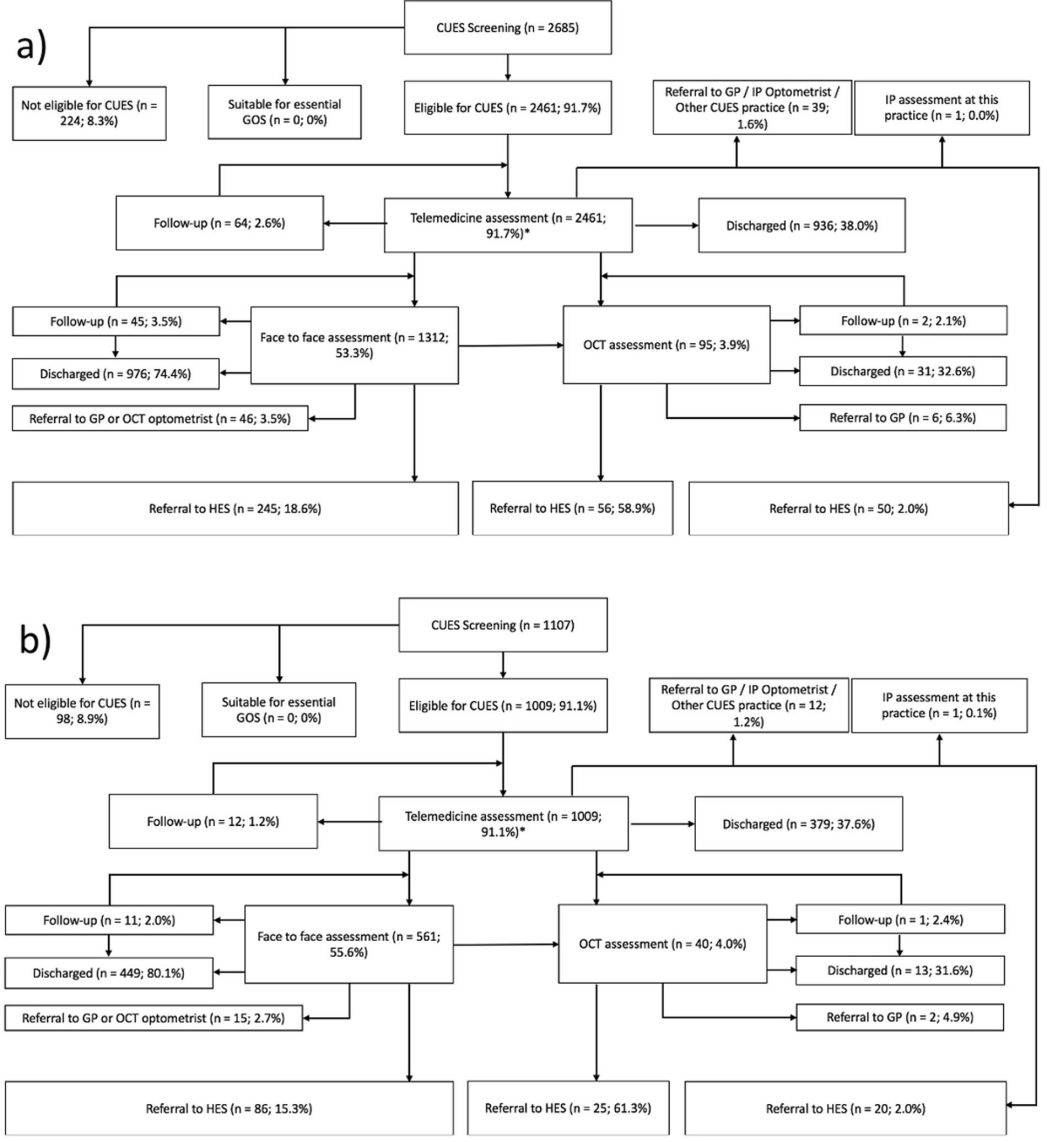
Flowchart depicting primary care data. Flowchart depicting primary care data for **a** GM CUES and **b** Manchester and Trafford CUES. **a** A breakdown of the primary care data at each stage for CUES in GM. **b** A breakdown of the primary care data at each stage for CUES in Manchester and Trafford. *Figures for outcomes of telemedicine assessments may not tally precisely due to selected patients having more than one follow-up appointment post face-to-face assessment or post OCT assessment. CUES COVID-19 Urgent Eyecare Service, GOS General Ophthalmic Services, GP General Practitioner, IP Independent Prescribing, OCT Optical Coherence Tomography, HES Hospital Eyecare Services.

Over the ~8-week period in June and July 2020, a total of 2685 patients underwent initial screening within GM CUES, with 1,107 of these patients being screened within CUES in MT. A flowchart depicting the primary care data for GM and MT is shown in Fig. 2. The proportion of cases that were initially deemed eligible for CUES and given a telemedicine appointment was similar in both GM (91.7%), as well as in MT (91.1%). In addition to this, a similar portion of people were given face-to-face appointments following a telemedicine appointment (GM—53.3%; MT—55.6%). The mean age of patients seen for initial screening within CUES was 52.0 years in GM, and 51.0 years in MT, with a higher proportion of these patients being female (GM—61.1%; MT—64.8%). Most patients had symptoms comprising painful, sore, red, sticky, watery, or itchy eye/s at screening (GM—58.3%, *n* = 1692; MT—60.3%, *n* = 668). A breakdown of primary care diagnoses for both telemedicine assessments and face-to-face assessments in GM are shown in Table 1. 13.0% of cases (131 out of 1009) seen in MT, and 14.3% in GM (351 out of 2461), over an 8-week period were eventually provisionally referred to secondary care HES.

**Table 1:** Table displaying frequency and percentage of all primary care diagnoses for both telemedicine assessments and face-to-face assessments in GM grouped anatomically.

Anatomical structure [list of diagnoses]		Total number of cases in GM (%)	
		Telemedicine assessment	Face to face assessment
Cornea	[Acanthamoeba keratitis; Acute corneal hydrops; Corneal abrasion; Corneal infection; Corneal ulcer; Dendritic ulcer; Foreign Body; Herpes keratitis; Keratoconus; Marginal keratitis; Microbial keratitis; Pterygium; Recurrent corneal erosions; Sterile infiltrates]	221 (9.0)	190 (14.2)
Eyelid	[Allergy—Eyelids; Blepharitis; Blepharospasm; Chalazion (Meibomian Cyst); Cyst of zeiss/Moll; Ectropion; Eczema; Entropion; External Hordeolum; Eyelid pathology—other; Herpes Zoster (shingles); Hordeolum (Stye); Ingrowing eyelash; Internal hordeolum; Lid Haematoma/bruise; Meibomian gland dysfunction; Ptosis; Sub conjunctival haemorrhage; Trichiasis]	435 (17.7)	196 (14.6)
Conjunctiva	[Allergic Conjunctivitis; Bacterial conjunctivitis; Concretions; Conjunctival abrasion; Conjunctivitis; Conjunctivitis medicamentosa; Pinguecula; Sub conjunctival haemorrhage; Viral conjunctivitis]	445 (18.1)	127 (9.5)
Uvea	[Anterior Uveitis; Iritis; Posterior Uveitis; Uveitis]	25 (1.0)	20 (1.5)
Vitreous	[Posterior Vitreous Detachment; Vitreous floaters—Signs of PVD or RD; Vitreous floaters—No signs of PVD or RD; Vitreous haemorrhage]	416 (16.9)	333 (24.9)
Lacrimal system	[Blocked tear duct; Dacrocystitis; Dry eye; Evaporative dry eye]	344 (14.0)	140 (10.5)
Retina and choroid	[BRVO/CRVO; Central serous retinopathy; CRAO/BRAO; Diabetic macular oedema; Diabetic retinopathy/maculopathy; Dry AMD; Epiretinal membrane; Macular hole; Macular oedema; Macula problem; Retinal detachment/tear/hole; Wet age-related macular degeneration]	178 (7.2)	72 (5.4)
Optic nerve and neurological	[Amaurosis Fugax; Angle closure glaucoma; Fourth nerve palsy; Glaucoma; Migraine visual aura; Myokymia; Nerve palsy— 3^rd^; Nerve Palsy—6^th^; Optic neuritis; Papilloedema; Sixth nerve palsy; Suspected temporal arteritis/GCA; Transient ischaemic attack]	149 (6.1)	83 (6.2)
Anterior chamber and aqueous	[Narrow angles; Ocular Hypertension; Traumatic hyphaemia]	0 (0.0)	5 (0.4)
Sclera	[Episcleritis; Scleritis; Sinusitis]	33 (1.3)	16 (1.2)
Orbit	[Preseptal cellulitis; Retrobulbar space occupying lesion]	8 (0.3)	4 (0.3)
Refractive and orthoptic	[Refractive Cause]	0 (0.0)	39 (2.9)
Lens	[Cataract; Posterior sub-capsular opacification]	20 (0.8)	45 (3.4)
Other	[Blunt Trauma; Chemical Injury; Contact dermatitis; Dermatitis; Diplopia; Eczema; Higher order field loss; Molluscum contagiosum; Other; Painful eyes; Possible horner’s syndrome; Red, Sore eye; Reduced vision; Retention cyst; Sinusitis; Sudden onset diplopia; Trauma; Unexplained vision loss; Unknown; Visual field defect; Visual hallucinations]	187 (7.6)	51 (3.8)
No pathology	[No ocular pathology identified]	0 (0.0)	17 (1.3)

### HES activity following referral from MT CUES

**Fig. 3:**
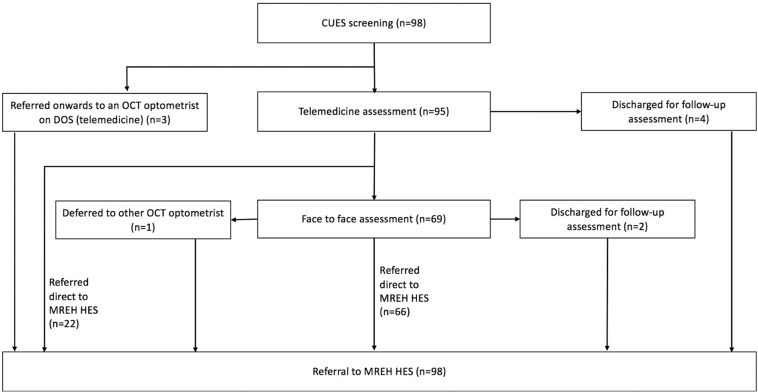
Flowchart for the pathway of the 98* (of 101) provisional CUES referrals received by MREH EED over the course of an 8-week period between June 2020 and August 2020. A breakdown of the primary care data at each stage of the process is reflected for Manchester CUES. *3 provisional referral forms could not be attained. CUES COVID-19 Urgent Eyecare Service, DOS Directory of Service, OCT Optical Coherence Tomography, MREH Manchester Royal Eye Hospital, HES Hospital Eyecare Services.

Over the course of the ~8-week period within June to August 2020, a total of 101 provisional referrals were received by MREH with provenance from CUES in MT. Out of these 101 provisional referrals, 98 referral forms (97.0%) were successfully retrieved to allow the analysis of referral quality (see Fig. 3).

### Quality of referral content

A detailed breakdown of the pathway for the 98 provisional CUES referrals received by MREH is shown in Fig. 3. All 98 referral forms (100.0%) included details of the initial screening, including time; date; the name of the practitioner; the name of optometric practice; GP practice name, address, and code; source of referral; consent details; whether the patient was a contact lens wearer and outcome of CUES screening. All 95 referral forms (100.0%) with attainable data telemedicine assessment data, included the date of the assessment, name of the clinician, and name of optometric practice. 86 referral forms (90.5%) indicated the outcome of the telemedicine assessment. However, none of these 95 referral forms (0.0%) detailed the clinician’s contact number, although the IT system does allow for advice and guidance to the referrer via eRS. Out of the 69 referral forms that contained primary care face-to-face assessment data, 62 (89.9%) clearly stated that the outcome of the face-to-face assessment was provisional referral to secondary care MREH.

### Accuracy of diagnoses

The single most common hospital diagnosis for the accepted MREH CUES referrals was uveitis (14.5%; 10 referrals); followed by AMD (11.6%; 8 referrals) and then BRVO/CRVO (10.1%; 7 referrals). Of the 101 provisional referrals received, 69 (68.3%) provisional referrals were accepted and 32 (31.7%) referrals were rejected. Out of the 69 accepted referrals, 8 (11.6%) were not able to be graded by the hospital clinicians, as either the primary or secondary care diagnoses were not attainable. 34 (49.3%) accepted referrals contained a primary care optometrist’s diagnosis graded as being in ‘agreement’ with the hospital clinician’s diagnosis and a further 5 (10.1%) of accepted referrals were graded being in ‘partial agreement’. A total of 22 (31.9%) of accepted referrals contained an optometrist’s diagnosis which was assigned as a ‘disagreement’ with the hospital diagnosis, albeit this disagreement does not infer that these referrals were unnecessary. Thus, of the 61 accepted referrals graded by the hospital clinicians, 39 (63.9%) were categorised as either being in ‘agreement’ or ‘partial agreement’. Those patients attending both telemedicine and face-to-face appointments in primary care were more likely to have either type of ‘agreement’ accuracy rating compared to those who attended telemedicine assessment only (chi-square test of independence *X*^2^ (1, *N* = 61) = 5.3, *p* = 0.02). Interestingly, of the 32 rejected referrals, 25 (78.1%) were rejected due to the patient’s condition not being deemed an emergency. In 2 (6.3%) out of the 32 rejected referrals, the patient attended the EED within 10 days of the date of initial non-acceptance of referral, and one of these cases was provisionally referred again through CUES at a later date, and in this case was accepted to be assessed in secondary care. The other case attended within 10 days and was treated for papilledema.

### EED activity

**Fig. 4:**
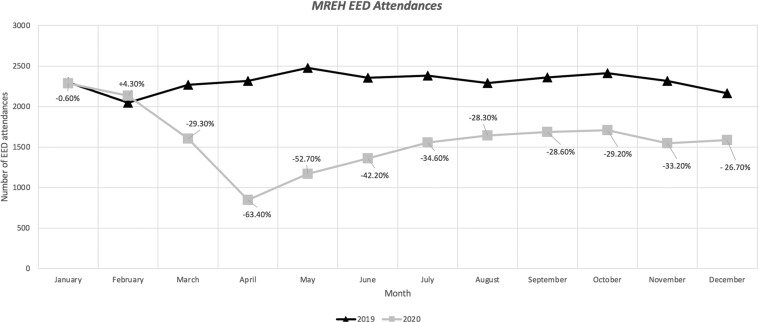
Graph displaying a summary of the number of MREH EED attendances from 2019 to 2020. The months of the year are listed on the horizontal axis. The total number of EED attendances is listed on the vertical axis. The percentage change from 2019 to 2020 for each month is displayed. EED: Emergency Eye Department.

The number of EED attendances from 2019 to 2020 is shown in Fig. 4. Phone calls received by the MREH EED team were recorded between 22^nd^ June and 11^th^ August 2020 inclusive (excluding on 5 dates where this data was unavailable). During this time, 420 records of telephone calls were recorded and signposted to either CUES, the MREH EED, or local hospitals/optometrist practices. In 56.0% (235 phone calls) the patient was advised to attend MREH EED and in 32.4% (136 phone calls) the patient was advised to see a CUES optometrist in the community, with the majority of those signposted to CUES relating to complaints of painful, red, sticky, sore, watery or itchy eye/s (41.2%; 56 phone calls).

## Discussion

A total of 2461 patient assessments within GM CUES were undertaken throughout the 8-week timeline of this evaluation, and the majority of these appeared to have self-referred to the CUES service (68.7%, *n* = 1844). Significantly, most CUES cases seen within primary care were also managed within primary care (85.7%; *n* = 2110), without the requirement to be referred to secondary care. The average age of patients who were referred to the initial screening of CUES was varied ranging from birth to 98.0 years and with a mean age of 52.0 years. Some 2461 cases (91.7% of those initially screened for the service) were seen by telemedicine assessments in GM, with 53.3% requiring a face-to-face consultation in primary care. It is evident that this primary care activity is contemporaneous with a significant change in the hospital’s EED activity, with a 29.3% reduction in activity occurring in March 2020, compared to March 2019. Thereafter, a further reduction in EED activity ensued during the lockdown in comparison to corresponding 2019 months, for example, April 2020 activity was reduced by −63.4% and May 2020 activity by −52.7%. Since May 2020, with the implementation of CUES and with lockdown restrictions easing at the time, the hospital’s EED activity sustained reduced activity compared to 2019 levels, arguably reflecting, at least in part, the implementation of CUES and the consequent availability of capacity to meet urgent eyecare demand within the community. The use of significant signposting to CUES following calls to the hospital’s EED nursing triaging appears to support this contention.

Despite CUES being designed to deal with urgent eyecare cases, only 13.0% of cases seen in MT (14.3% overall in GM) required provisional referral to secondary care. Even here, in the case of MREH and activity within MT CUES, a proportion of these referrals (31.7%; *n* = 32) were rejected with advice and guidance, largely due to the patients’ condition not being deemed to be sufficiently urgent for EED assessment, highlighting the potential for the effectiveness of the service to be further improved. Indeed, there is scope for improvement, since the implementation of IP optometric assessment was delayed pending commissioner medicines management approval and was not therefore part of the current evaluation. This arm of CUES is likely to further decrease the number of provisional referrals to HES, i.e. with an enhanced case-mix being manageable in primary care. Interestingly, as reported in their evaluation of MECS, Konstantakopoulou et al. noted a 19.3% HES referral rate from MECS, higher than our referral figures for CUES [6]. This lower referral rate from CUES may be due in part to the increase in remote consultations and the focused management of patients in primary care during COVID19 and/or a shift in emphasis in the expected scope of practice within primary care services. A study undertaken by Siempis found that the most common UK urgent eyecare diagnosis was conjunctivitis, followed by: foreign body in the eye, a cyst, and then a dry eye [12]. In comparison, the most common hospital diagnoses for accepted CUES referrals were uveitis, AMD, and BRVO/CRVO, representative of a more urgent case mix, and reflective of the service achieving its aims of keeping the more benign cases within CUES itself away from the HES. Indeed, this suggestion would appear to be borne out by a previous local audit of over 500 MREH EED diagnoses in 2017, which saw conjunctivitis and ocular surface-related diagnoses as the most common presentations [13].

In this evaluation of CUES, 23.9% of accepted referrals were ultimately categorised as being cases that may not have needed referral (i.e. based upon a comparison of primary care and secondary care diagnoses). Given the case-mix of patients seen within CUES and the use of telemedicine to reduce face-to-face contact in as many assessments as feasible, it is inevitable that a number of cases may ultimately be deemed to be false positives; however, this proportion of cases need to be seen within the context of all of the cases seen within CUES and managed within primary care and not referred in. Interestingly, patients who attended a face-to-face assessment in CUES were more likely to have a diagnosis in agreement/partial agreement with that of the secondary care examination, compared to patients who were referred to HES directly from a telemedicine assessment, a finding that can reasonably be argued to have been expected.

Cameron et al. demonstrated that the use of electronic referrals to the HES, with the ability to attach images, has already helped reduce the number of unnecessary referrals [14]. However, with the added pressure of the pandemic and the demand for face-to-face interaction to be reduced for the foreseeable future, the need to decrease unnecessary referrals is stronger than ever. In our view, the present evaluation of CUES reflects a very promising start for this primary care service, and with the benefit of interlinking OPERA and advice and guidance around referrals therein, there is a template for future services to follow. It needs to be acknowledged, however, that there are some limitations to our first evaluation of CUES. First, we have not, to date, examined CUES for possible ‘false negatives’, i.e. those non-referred cases seen within primary care who may have actually required referral. We plan to undertake such an evaluation in the future. Second, we have not reported on patient satisfaction data and there are plans to routinely collate this feedback as part of the primary care service. Furthermore, as noted above, in most areas of GM where CUES was established during April-August 2020, the facilitation of IP optometrists to prescribe in the community was not realised at the time of this evaluation. Interestingly, a number of those accepted referrals seen in MREH’s EED could have been appropriately managed in primary care by an optometrist with IP had the prescribing arrangements been established. It is reasonable to speculate that a higher proportion of optometrists trained and accredited in IP (as well as having the relevant experience with the appropriate case-mix of patients) will be key to delivering enhancements in CUES. The role of optometrists with IP accreditation within secondary care was recently investigated by Todd et al. [15] who showed that IP optometrists with significant experience were able to attain a high level of agreement in clinical decision making with consultants in EED. Further work is necessary to establish if their very positive findings might be realisable within primary care. Finally, a further limitation is arguably the single centre data we present for referrals into secondary care (MREH EED), although our primary care data may be considered to be generalisable, arising as it does from large scale activity across multiple CCGs within the Greater Manchester conurbation, and offering evidence in support for CUES growing in scale nationally [9].

In conclusion, this early evaluation of CUES demonstrates a high level of activity in CUES in primary care alongside a sustained reduction in patient numbers attending secondary care acute services. Although developed for the unique situation of COVID-19, it is evident that there is a requirement for CUES to evolve into a long-term primary care service [16]. Further evaluation will be important in refining the service, including how best to optimise the role that the advice and guidance element within the pathway can provide at the interface of primary and secondary care, as well as investigating the enhancements that can be conferred to CUES by expanding the critical mass of those practitioners trained and accredited in IP.
